# Chlorogenic acid ameliorated allergic rhinitis-related symptoms in mice by regulating Th17 cells

**DOI:** 10.1042/BSR20201643

**Published:** 2020-11-02

**Authors:** Zhaohui Shi, Weihong Jiang, Xiaodong Chen, Min Xu, Jian Wang, Yubin Lai, Dingjun Zha

**Affiliations:** 1 Department of Otolaryngology-Head and Neck Surgery, Xijing Hospital, Air Force Millitary Medical University, Xi’an, Shaanxi Province, China; 2 Department of Otolaryngology-Head and Neck Surgery, Xiangya Hospital, Central South University, Changsha, Hunan Province, China

**Keywords:** chlorogenic acid, allergic rhinitis, CD4+IL-17+Th17 cells, CD4+T cells, Th17

## Abstract

Allergic rhinitis (AR) is a non-infectious chronic inflammatory disease of nasal mucosa provoking T helper cell (Th) 17 response. Chlorogenic acid (CGA), one of the most abundant polyphenol compounds in various agricultural products, possesses antiviral, anti-inflammatory, and antibacterial properties. However, the effect of CGA on AR is unclear. Thus, our study explored the effect of CGA in modulating AR-related symptoms and immunoreaction, especially Th17 response. AR mice were induced by ovalbumin (OVA) administration and further treated with CGA or dexamethasone (Dex). The frequencies of rubbing and sneezing of AR mice were recorded. Histopathological analysis of nasal mucosa was conducted by Hematoxylin–Eosin and Periodic acid–Schiff stainings. The serum and nasal mucosa levels of OVA-immunoglobulin (Ig)E, interferon (IFN)-γ, retinoic acid-associated nuclear orphan receptor (ROR)-γt, and interleukin (IL)-17A were measured by enzyme-linked immunosorbent assay, quantitative reverse-transcription polymerase chain reaction (qRT-PCR), or Western blot. The ratio of CD4^+^IL-17^+^Th17 cells to CD4^+^ T cells in peripheral blood of AR mice was assessed by flow cytometer. CGA diminished the frequencies of rubbing and sneezing of AR mice in a concentration-dependent manner. CGA attenuated histopathological abnormalities and decreased goblet cell number in nasal mucosa of AR mice. CGA decreased the serum levels of OVA-IgE, ROR-γt, and IL-17A, while increasing the serum level of IFN-γ in AR mice. Meanwhile, CGA decreased the ratio of CD4^+^IL-17^+^Th17 cells to CD4^+^T cells in peripheral blood and the mRNA and protein levels of IL-17A and ROR-γt in AR mice. CGA ameliorated AR-related symptoms in mice by regulating Th17 cells, which could be a candidate for the treatment of AR.

## Introduction

Allergic rhinitis (AR), an allergen-caused inflammation in nasal airway is characterized by nose rubbing, sneezing, rhinorrhea, and nasal obstruction, and it can also be a risk factor for diseases like asthma, rhinosinusitis, and adenoid hypertrophy. After being exposed to harmless allergen, atopic individuals are predisposed to be subjected to a chronic inflammatory response implicating activity of immune cells and cytokines and immunoglobulin E (IgE)-mediated release of proinflammatory factors cytokines in nasal mucosa [1,2,3]. Over 40% of the people worldwide were substantially affected by AR in their sleep, school, work, and social life [4].

The pathogenesis of AR is generally accepted as imbalanced T helper cells (Th) 1/Th2 immunological reaction mechanism [5,6]. Th1 and Th2, two subsets of helper T cells with different functions are important in regulating immune response [7,8]. During AR, CD4^+^ T cells are driven to evolve into Th2, which results in eosinophilia and Th2 over-differentiation [7,9]. Therefore, previous therapy for AR has emphasized on pro-Th1 cell differentiation for regaining the balance of Th1/Th2 [6]. However, this therapy was not effective and even caused further problems such as acute lung pathology [10]. Recent findings show that both Th17 cells and pTreg cells whose precursor cells are also CD4 T play vital roles in autoimmunity response that can be induced by AR [11]. Th17 cells can cause autoimmunity and inflammation, however which can be suppressed by Th17 cells to thus attain immune homeostasis [12]. Interleukin (IL)-6 or IL-21 facilitates differentiation of CD4^+^ T cells into Treg cells, whereas CD4^+^ T differentiates into Treg cells without IL-6 or IL-21 [13,14]. Th17 cells are found to generate proinflammatory cytokines like IL-17, IL-22, and IL-23 to aggravate inflammation and recruit neutrophils at the site of infection [12]. In turn, a series of cytokines produced during Th-cell differentiation to induce AR can impact on CD4^+^ T cells fate to further affect Th17 differentiation [15,16]. Hence, Th17, as host defense against mucosal inflammation [16], has potential to be a novel target of immunity therapy for AR.

Chlorogenic acid (CGA), also known as 3-Caffeoquininic acid is a hemihydrate presented in acicular crystals that is widely found in many kinds of Chinese herbal medicines, such as honeysuckle, houttuynia cordata and honeysuckle [17]. Owning that it possesses multiple biological activities such as antibacterial, antiviral, anti-inflammatory, cardiovascular protection, and immunomodulatory, CGA has been widely studied in serious research *in vitro* and *in vivo* [18]. For instance, CGA has anti-inflammatory effect on *Escherichia coli*-induced inflammation of sheep endometrial epithelium cells [19], the anti-inflammatory effect of CGA on lipopolysaccharide-induced inflammatory response in dairy mammary epithelial cells was also reported by Gao et al. [20]; CGA also ameliorates the innate immunity-related endotoxic shock and acute liver injury [21]. However, how CGA acts on allergic inflammation and immune response in AR has not been fully elucidated.

Thus, the present study sought to reveal the potential value of CGA as an anti-allergic and immunomodulatory therapy for AR.

## Materials and methods

### AR mouse model establishment and grouping

Thirty-six BALB/c mice aged 6–8 weeks, weighing 22–30 g, were purchased from Slac Laboratory Animal Co. Ltd (Shanghai, China). All the mice were maintained in pathogen-free conditions at 19–24°C, in 50% humidity, with 12/12-h light to night circle and given unrestricted access to ample food and water. Before being subjected to experimentation, the mice were acclimatized for at least 7 days.

Ovalbumin (OVA, S7951), CGA (C_16_H_18_O_9_, C3878, purity ≥ 95%), and dexamethasone (Dex, C_22_H_29_FO_5_, D4902, purity ≥ 97%) were all purchased from Sigma–Aldrich LLC (St. Louis, MO, U.S.A.).

The mice were randomly divided into six groups (*n*=6 per group), which were Control group, OVA group, OVA+CGA-L (low) group, OVA+CGA-M (medium) group, OVA+CGA-H (high) group, and OVA+DEX group. The mice in the latter five groups were sensitized via intraperitoneal injection of 100 μg OVA plus 1 mg alum (237086, Sigma–Aldrich, U.S.A.) at days 1, 5, and 8 and were challenged via nasal drug delivery of 10 μg of 10% OVA solution diluted by normal saline from days 22 to 28. Fortunately, all mice were successfully induced into AR mice by OVA. Starting from days 16 to 28, the mice in the OVA+CGA-L, OVA+CGA-M, and OVA+CGA-LH groups underwent oral gavage of 200 μl CGA (50, 100, and 200 mg/kg), respectively. OVA+DEX group in which the mice were treated with intragastric infusion of Dex (2.5 mg/kg) was set as the positive control. The mice in the control group were free of sensitization, challenge, and drug treatment. On day 29, all the mice were anesthetized via intraperitoneal injection of 2% pentobarbital sodium (P-010, Sigma–Aldrich, U.S.A.) at a dose of 50 mg/kg and were euthanized, 24 h after the last OVA challenge. Mice blood collected was added with heparin sodium (H3149, Sigma–Aldrich, U.S.A.) for detection of Th17 differentiation. Mouse nasal mucosa taken was fixed in 4% paraformaldehyde (P6148, Sigma–Aldrich, U.S.A.) for histopathological examination or frozen at −80°C by liquid nitrogen for being used in quantitative reverse-transcription polymerase chain reaction (qRT-PCR) and Western blot.

### Hematoxylin–Eosin staining

For observing the histopathological changes in nasal mucosa after CGA or Dex intervention, a 4-cm-thick mouse nasal mucosa was fixed in 4% paraformaldehyde (P6148, Sigma–Aldrich, U.S.A.), dehydrated by ethanol (E7023, Sigma–Aldrich, U.S.A.), blocked in paraffin (327204, Sigma–Aldrich, U.S.A.), and sliced. Then, the sliced mouse nasal mucosa was soaked in xylene (95670, Sigma–Aldrich, U.S.A.) for dewaxation. Gradient ethanol 100, 90, 80, and 70% was used to rehydrate mouse nasal mucosa slices. Mouse nasal mucosa slices were stained by Hematoxylin (H3136, Sigma–Aldrich, U.S.A.) for 15 min, differentiated by being soaked in 5% acetic acid (A6283, Sigma–Aldrich, U.S.A.), and immersed in distilled water for developing blue. Next, mouse nasal mucosa slices were stained by Eosin (E4009, Sigma–Aldrich, U.S.A.) for 10 min. Gradient ethanol 70, 80, 90, and 100% was used to dehydrate mouse nasal mucosa slices. After soaking in xylene twice, mouse nasal mucosa slices were sealed by Canada balsam (60610, Sigma–Aldrich, U.S.A.) and observed using an optical microscope (M3800, Fisher Scientific, Waltham, MA, U.S.A.).

### Periodic acid–Schiff staining

Counting of goblet cells was achieved after CGA or Dex intervention by Periodic acid–Schiff (PAS) staining according to PAS assay kit (G1286, Solarbio, Beijing, China). Mice nasal mucosa slices were processed with Periodic Acid for 10 min. After rinsing with water, Schiff’s solution was added dropwise on the mice nasal mucosa slices and stained for 15 min. Later, Mayer Hematoxylin was used to counterstain mice nasal mucosa slices for 3 min. Following washing by water, the slices were dehydrated, transparentized, and sealed for observation under an optical microscope (M3800, Fisher Scientific, Waltham, MA, U.S.A.).

### Enzyme-linked immunosorbent assay

After CGA or Dex intervention, mice blood collected from the tail was centrifuged at 1000×***g*** for 10 min for separating serum. OVA-IgE, interferon (IFN)-γ, retinoic acid-associated nuclear orphan receptor (ROR)-γt, and IL-17A in the serum were detected by IgE (KGEMC117-1), IFN-γ (KGEMC101g-1), and IL-17A (KGEMC008-1) ELISA kits purchased from Keygentec (Nanjing, China) and ROR-γt (EM8104) ELISA kit purchased from FineTest (Wuhan, China) in accordance with manufacturers’ instructions. Briefly, 100 μl of sample serum was added into each well of the experimental slabs and incubated at 36°C for 90 min. After the slabs were washed with detergent thrice, the sample was added with 100 μl of biotinylated antibodies and incubated at 36°C for 1 h. Then, 100 μl of enzyme-labeled streptavidin was added into the serum for incubation at 36°C for 30 min in the darkness. A total of 50 μl of rendering agent liquid A and 50 μl of rendering agent liquid B were mixed for 30 s and added into the serum. Color was developed at 37°C for 15 min in the darkness; 0.5 ml of 2 M H_2_SO_4_ was used to terminate the reaction. Within 3 min after reaction termination, optical density at the wavelength of 450 nm was measured via a microplate reader (Infinite M200 PRO, Tecan Austria GmbH, Austria).

### Flow cytometry

After CGA or Dex intervention, 100µL of collected mice blood was processed with 30 μl of heparin sodium, added with 100 μl of EDTA (EDS, Sigma–Aldrich, U.S.A.) and centrifuged at 2000×***g*** for 20 min with 300 μl of lymphocytes separating fluid (P8610, Solarbio, Beijing). Later, peripheral blood mononuclear cell layer was taken, cultured in RPMI 1640 (A4192301, Thermo Fisher, Waltham, MA, U.S.A.) containing 15% fetal bovine serum (1921005PG, Thermo Fisher, U.S.A.) and incubated with CD4-APC (MA5-17447, Thermo Fisher, U.S.A.) at 4°C for 30 min in the darkness. After washing with phosphate buffer saline (PBS, 28348, Thermo Fisher, U.S.A.) and centrifuging at 1000×***g*** for 10 min, blood sample was incubated with Fixation Reagent (GAS003, Thermo Fisher, U.S.A.) at room temperature for 20 min for fixation. Then, blood sample was added with Permeabilization Reagent (GAS003, Thermo Fisher, U.S.A.) and centrifuged at 2000×***g*** for 10 min for cell rupture. Then, IL-17-FITC (A15377, Thermo Fisher, U.S.A.) was added into blood sample and incubated at 4°C for 45 min in the darkness. Following washed by and resuspeneded by Permeabilization Reagent, blood sample was transferred on to a flow cytometer (FASCAria™, BD Bioscience, MA, U.S.A.) and analyzed by FlowJo software (version 7.6.1, Treestar, U.S.A.).

### qRT-PCR

Total mRNA in mice nasal mucosa was isolated by TRIzol LS Reagent (10296010, Thermo Fisher, U.S.A.). mRNA extracted by chloroform (48520-U, Sigma–Aldrich, U.S.A.) was precipitated by isopropanol (I9516, Sigma–Aldrich, U.S.A.), followed by washing with ethanol, and solubilized in RNase-free water (10977023, Thermo Fisher, U.S.A.). Synthesis of cDNA template of the extracted mRNA was done using SuperScript IV reverse transcriptase (18090010, Thermo Fisher, U.S.A.). Amplification of cDNA in qRT-PCR was conducted using PowerUp SYBR Green Master Mix (A25742, Thermo Fisher, U.S.A.) on Applied Biosystems 7500 Real-Time PCR System (4377354, Thermo Fisher, U.S.A.). The cycling conditions were: 50°C Uracil-DNA Glycosylase enzyme activation for 2 min, at 95°C pre-denaturation for 2 min, followed by 40 cycles of 95°C denaturation for 15 s, 60°C annealing for 60 s, and 60°C extension for 60 s. The primers used in the reaction were: ROR-γt (forward: 5′-GACCCACACCTCACAAATTGA-3′, reverse: 5′-AGTAGGCCACATTACACTGCT-3′), IL-17A (forward: 5′-TTTAACTCCCTTGGCGCAAAA-3′, reverse: 5′-CTTTCCCTCCGCATTGACAC-3′), and glyceraldehyde-3-phosphate dehydrogenase (GAPDH; forward: 5′-AGGTCGGTGTGAACGGATTTG-3′, reverse: 5′-TGTAGACCATGTAGTTGAGGTCA-3′). Automated threshold analysis of the cycler was conducted to determine threshold cycle (*C*_t_) values. The relative expression levels of ROR-γt and IL-17A were normalized to that of GAPDH. Results were expressed as fold change by 2^−ΔΔ*C*_t_^ method [22].

### Western blot

Total protein isolated from mice nasal mucosa was lysed by RIPA Buffer (89900, Thermo Fisher, U.S.A.) and its concentration was determined using a BCA protein assay kit (A53227, Thermo Fisher, U.S.A.). A 35-μl aliquot of protein as well as 5 μl of marker (PR1910, Solarbio, China) was laid on to a 12% sodium dodecyl sulfate/polyacrylamide gel electrophoresis (SDS/PAGE gel; P0053A, Beyotime, Shanghai, China) and was electrophoresed. The separated proteins were transferred on to a polyvinylidene difluoride (PVDF) membrane (P2438, Sigma–Aldrich, U.S.A.). The membrane was blocked with 5% skimmed milk for 60 min at room temperature immersed in Tris Buffered Saline and 1% Tween 20 (TBST, TA-125-TT, Thermo Fisher, U.S.A.). Later, the membranes were incubated overnight at 4°C overnight with the following primary antibodies diluted in PBS: ROR-γt (ab232516, 58 kDa, 1:1000, Abcam, Cambridge, MA, U.S.A.), IL-17A (ab150719, 17 kDa, 1:1000, Abcam, Cambridge, U.S.A.), and GAPDH (ab181602, 37 kDa, 1:10000, abcam, U.S.A.). Following washing with TBST, the membranes were incubated with secondary antibody: Goat Anti-Rabbit IgG H&L (HRP) (ab205718, 1:2000, Abcam, U.S.A.) for 1 h, at room temperature. Then immunoreactive bands were developed on an imaging System (iBright™ CL1500, A44240, Thermo Fisher, U.S.A.) using enhanced chemiluminescence reagent kit (WP20005, Thermo Fisher, U.S.A.). Quantification of the density of the immunoreactive bands was performed with ImageJ 1.52s software (National Institutes of Health, Maryland, U.S.A.).

### Statistical analysis

Data were presented as means ± SD and analyzed using SPSS 16.0 software (IBM, Armonk, NY, U.S.A.). One-way ANOVA was used to analyze the differences among more than three groups, followed by Tukey’s test. *P*<0.05 was considered as statistically significant. All experiments were performed for at least three times.

## Results

### OVA ameliorated the symptoms of rubbing and sneezing in AR mice

**Figure 1:**
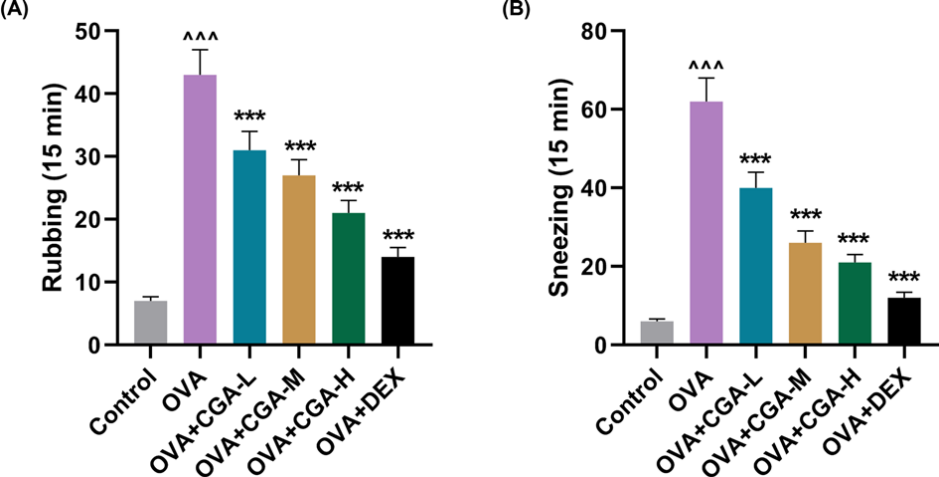
OVA ameliorated the symptoms frequencies of rubbing and sneezing in AR mice (**A**) The frequency of rubbing behavior of AR mice, treated with different concentrations (low, medium, and high) of CGA or Dex in 15 min. (**B**) The frequency of sneezing behavior of AR mice, treated with different concentrations (low, medium, and high) of CGA or Dex in 15 min. ^∧∧∧^*P or* ***P<0.001; ^∧^ vs. Control; * vs. OVA (L, low concentration of CGA; M, medium concentration of CGA; H, high concentration of CGA).

For investigating the anti-allergic effect of CGA, AR model was established on mice and the frequencies of rubbing and sneezing of AR mice were recorded following OVA challenge. OVA-induced AR mice displayed significantly increased frequencies of rubbing and sneezing, compared with control mice (Figure 1A,B, *P*<0.001). The incidences of rubbing and sneezing were evidently reduced in AR mice after being forcefully fed with CGA, compared with AR mice without gavage (Figure 1A,B, *P*<0.001). Notably, as the concentration of CGA for gavage was increasing, the frequencies of rubbing and sneezing were reduced. Dex, a glucocorticoid having been proven effective as anti-allergic drug in AR [23], was used as positive control to reflect to what degree CGA could suppress rubbing and sneezing in AR mice. Dex gavage most significantly reduced the frequencies of rubbing and sneezing in AR mice, compared with AR mice without gavage (*P*<0.001), while gavage of high concentration of CGA (200 mg/kg) showed less anti-rubbing and sneezing effect, as evidenced by the frequencies of rubbing and sneezing (Figure 1A,B, *P*<0.001). These results indicated that CGA ameliorated the symptoms of rubbing and sneezing in AR mice.

### CGA reduced histopathological abnormalities and goblet cell number in nasal mucosa of AR mice

**Figure 2:**
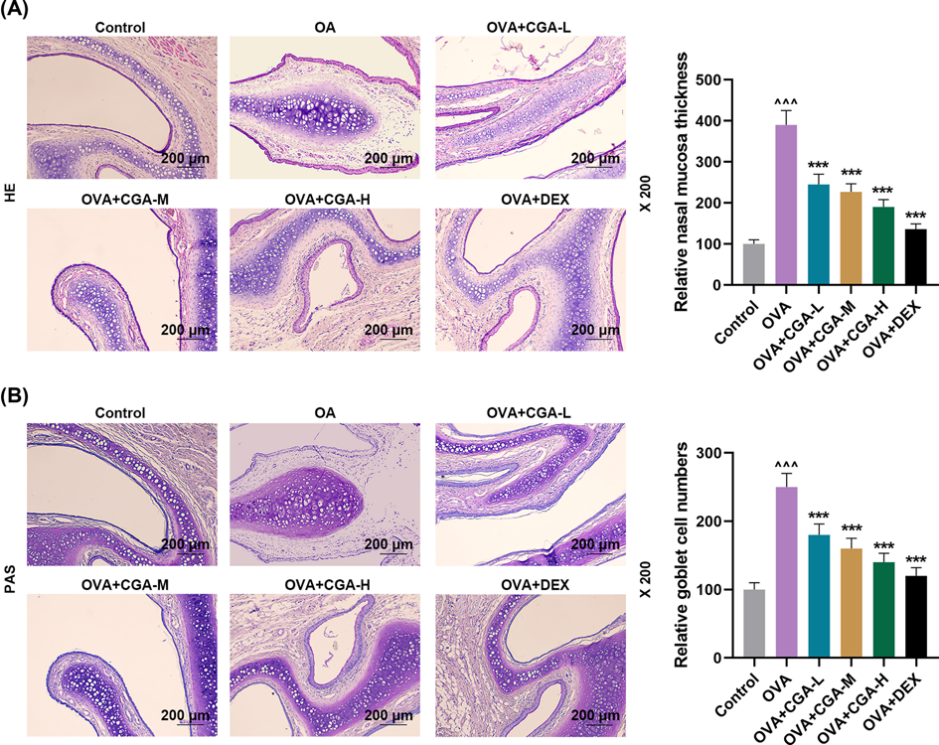
CGA reduced histopathological abnormalities and goblet cell number in nasal mucosa of AR mice (**A**) Representative photos of histopathological changes of nasal mucosal thickness of mice with AR, treated with different concentrations (low, medium, and high) of CGA or Dex, reflected by Hematoxylin–Eosin staining (magnitude: 200×; scale: 200 μm); the semi-quantative analysis of the images is also shown on the right. (**B**) Representative photos of goblet cells in nasal mucosal thickness of mice with AR, treated with different concentrations (low, medium, and high) of CGA or Dex, reflected by PAS staining (magnitude: 200×; scale: 200 μm); the semi-quantative analysis of the images is also shown on the right.^ ∧∧∧^*P* or ***P<0.001; ^∧^ vs. Control; * vs. OVA (L, low concentration of CGA; M, medium concentration of CGA; H, high concentration of CGA). ^∧∧∧^*P* or ***P<0.001; ^∧^ vs. Control; * vs. OVA.

For exploring how CGA impacted on OVA-induced histopathological changes, nasal mucosa thickness and goblet cell number in nasal mucosal of AR mice was observed. Marked hypertrophy of nasal mucosa with evident infiltration of eosinophils, enhanced mucus secretion, and increased number of goblet cell was shown in OVA-induced AR mice, compared with control mice (Figure 2A,B). Gavage of CGA and Dex reduced nasal mucosa thickness, eosinophils infiltration, mucus secretion, and goblet cell number in AR mice (Figure 2A,B). More specifically, the higher concentration of CGA AR mice was fed with the thinner thickness of nasal mucosa, the less degree of eosinophils infiltration and mucus secretion, and the smaller number of goblet cell were in nasal mucosal of AR mice (Figure 2A,B). The results suggested that CGA could reverse the AR-related histopathological changes in nasal mucosa.

### CGA reversed the effect of OVA on the serum levels of OVA-IgE, IFN-γ, ROR-γt, and IL-17A

**Figure 3:**
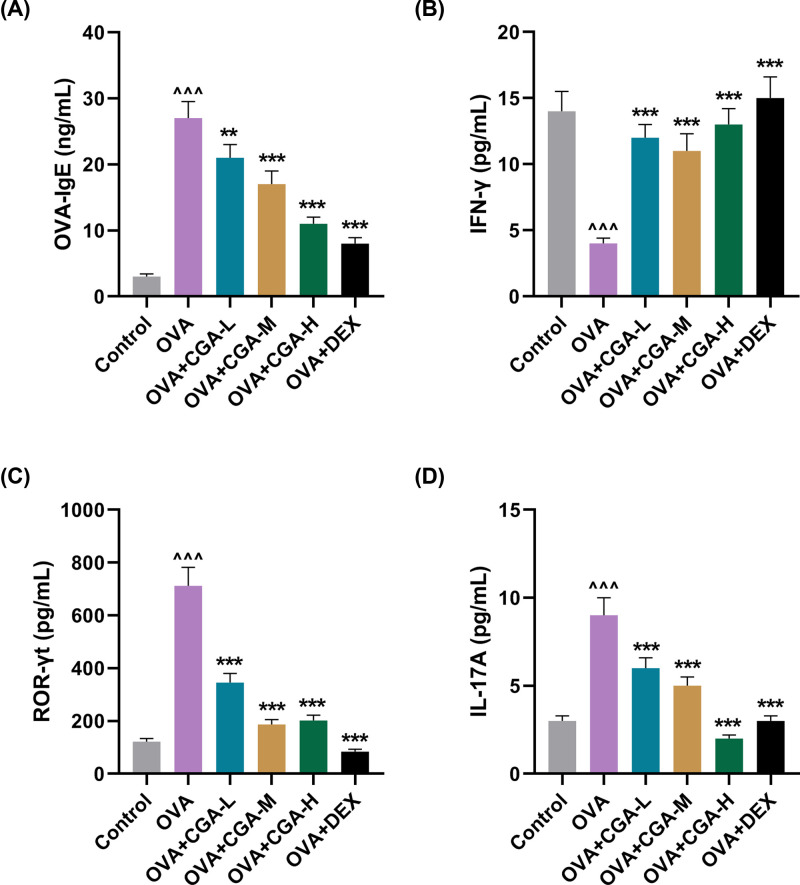
CGA reversed the effect of OVA on the serum levels of OVA-IgE, IFN-γ, ROR-γt, and IL-17A (**A**) The serum level of OVA-IgE was measured by ELISA in AR mice treated with different concentrations (low, medium, and high) of CGA or Dex. (**B**) The serum level of IFN-γ was measured by ELISA in AR mice treated with different concentrations (low, medium, and high) of CGA or Dex. (**C**) The serum level of ROR-γt was measured by ELISA in AR mice treated with different concentrations (low, medium, and high) of CGA or Dex. (**D**) The serum level of IL-17A measured by ELISA in AR mice treated with different concentrations (low, medium, and high) of CGA or Dex. **P<0.01, ^∧∧∧^*P* or ***P<0.001; ^∧^ vs. Control; * vs. OVA (L, low concentration of CGA; M, medium concentration of CGA; H, high concentration of CGA).

To unveil the underlying mechanism of the anti-allergic effect of CGA, Th cell differentiation-related serum levels of IgE, IFN-γ, ROR-γt, and IL-17A in AR mice was measured. The levels of OVA-IgE, ROR-γt, and IL-17A were up-regulated in OVA-induced AR mice, compared with that in control mice (Figure 3A–D, *P*<0.001). Gavage of low concentration of CGA lowered the serum level of OVA-IgE, ROR-γt, and IL-17A in AR mice, compared with that in AR mice without gavage. (Figure 3A, *P*<0.01, *P*<0.01, *P*<0.01, respectively). Certainly, gavage of medium or high concentration of CGA or Dex also markedly lowered the levels of OVA-IgE, ROR-γt, and IL-17A in AR mice, compared with AR mice without gavage (Figure 3A–D, *P*<0.001). However, the serum level of IFN-γ was down-regulated in OVA-induced AR mice, compared with that in the control mice (Figure 3B, *P*<0.001). Gavage of low, medium, or high concentration of CGA or Dex reversed the serum level of IFN-γ that was down-regulated by OVA administration (Figure 3B, *P*<0.001). These results suggested that the anti-AR effect of CGA may be highly associated with regulation of the levels of OVA-IgE, IFN-γ, ROR-γt, and IL-17A in AR mice.

### CGA regulated OVA-induced Th17 differentiation

**Figure 4:**
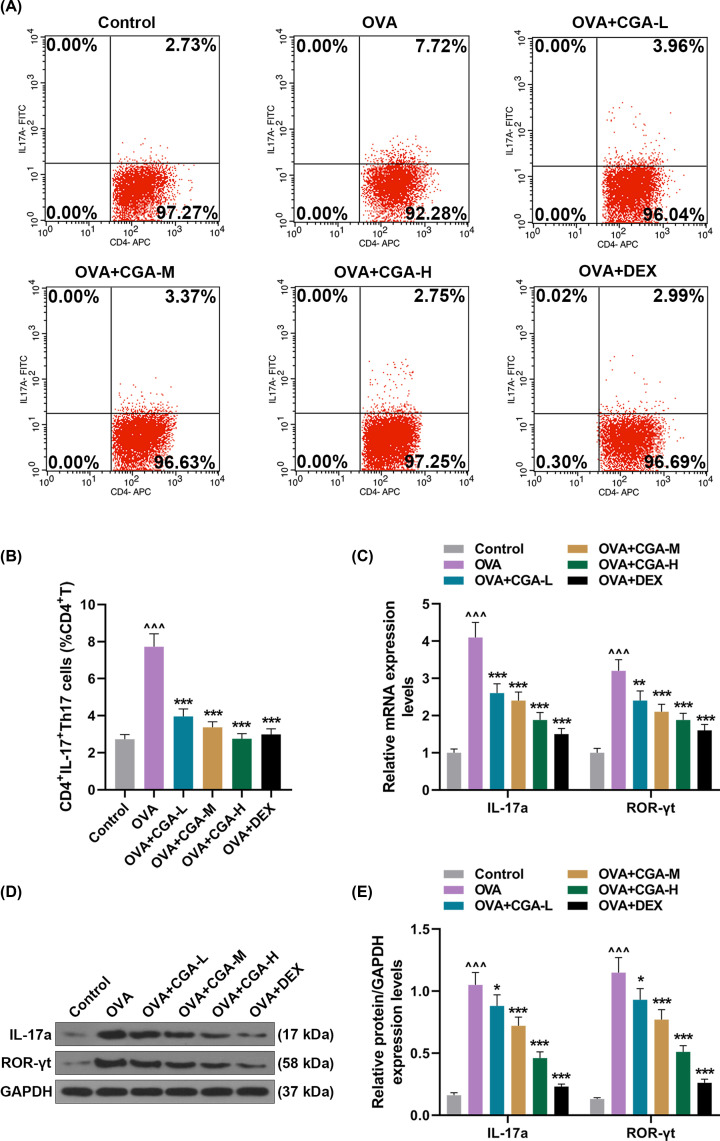
CGA regulated OVA-induced Th17 differentiation (**A**) Representative pictures of the testing results of flow cytometer for the ratio of CD4^+^IL-17^+^Th17 cells to CD4^+^ T cells in peripheral blood of AR mice treated with different concentrations (low, medium, and high) of CGA or Dex. (**B**) The ratio of CD4^+^IL-17^+^Th17 cells to CD4^+^ T cells in peripheral blood of AR mice treated with different concentrations (low, medium, and high) of CGA or Dex was assessed by flow cytometer. (**C**) The mRNA expressions of IL-17a and ROR-γt in AR mice treated with different concentrations (low, medium, and high) of CGA or Dex were analyzed by qRT-PCR, with GAPDH serving as a reference gene. (**D**) Pictures of the protein bands of IL-17a, ROR-γt, and GAPDH. (**E**) The protein expressions of IL-17a and ROR-γt in AR mice treated with different concentrations (low, medium, and high) of CGA or Dex were analyzed by Western blot, with GAPDH serving as a reference gene. *P<0.05, **P<0.01, ^∧∧∧^*P* or ***P<0.001;^ ∧^ vs. Control; * vs. OVA (L, low concentration of CGA; M, medium concentration of CGA; H, high concentration of CGA CD4^+^ T, surface antigen differentiation cluster 4 receptor^+^ T lymphocyte).

The differentiation and proliferation of Th17 cells is widely recognized to involve the pathogenesis of autoimmune diseases and allergic diseases [24]. Thus, to assess the effect of CGA on Th17 response, the Th17 differentiation-related indicators were detected. Large number of CD4^+^IL^−^17^+^Th17 cells were spotted in peripheral blood of AR mice, compared with control mice (Figure 4A,B, *P*<0.001), while gavage of low, medium, or high concentration of CGA or Dex uniformly suppressed increase in CD4^+^IL^−^17^+^Th17 cells in AR mice (Figure 4A,B, *P*<0.001). Meanwhile, the mRNA and protein expressions of ROR-γt and IL-17A were facilitated in AR mice, compared with control mice (Figure 4C–E, *P*<0.001). In contrast, the mRNA and protein expressions of IL-17A were inhibited after AR mice were forcefully fed with low concentration of CGA, compared to AR mice without gavage (Figure 4C–E, *P*<0.001, *P*<0.05, respectively). Similarly, the mRNA and protein expressions of ROR-γt were inhibited by gavage of low concentration of CGA, compared with AR mice without gavage (Figure 4C–E, *P*<0.01, *P*<0.05, respectively). Certainly, gavage of medium or high concentration of CGA or Dex repressed the mRNA and protein expressions of ROR-γt and IL-17A that were facilitated in AR mice without gavage (Figure 4C–E, *P*<0.001). These results showed that the anti-AR effect of CGA may be exerted via suppressing Th17 differentiation and inflammation.

## Discussion

AR a widely encountered allergic disease is mainly reflected in inflammation at the nasal airways, accompanied with Th2 cell overproduction, eosinophilia, goblet cell hyperplasia, and allergen-specific IgE production [25,26]. Previous study believed that the imbalance of Th1/Th2 cells was the predominant pathogenesis of AR [27]. Recently, Th17/Treg cells sharing the same precursor cell (CD4^+^ T cells) as Th1/Th2 cells were discovered to be typically unbalanced during allergic inflammation [28]. Th17 cells induced by a Th2 cytokine (IL-6) are recognized as a pivotal player in modulating autoimmune response and combating bacterial infections [29,30]. Study found that abnormal differentiation of Th17 cells caused by aberrant production of IL-6 and TGF-β is considered as a primary factor for AR [31]. Therefore, focusing on Th17 cell differentiation might well contribute to discover an immunomodulatory approach for alleviating AR.

CGA, a polyphenolic compound can exert anti-arthritic effects via inhibiting nitric oxide production and suppressing T-cell proliferation [32]. CGA is recorded to facilitate lymphocyte proliferation specific to antigens to favor influenza virus-specific antibodies generation using mononuclear cells in human peripheral blood [33]. As AR is closely associated with nasal inflammation triggered by immune cell-induced cytokine infiltration, we applied different concentrations of CGA on OVA-induced AR mice. Dex, glucocorticoids, is confirmed to be effective on suppressing nose rubbing, sneezing, and nasal discharge and mitigating allergic inflammation [34], was used as positive control in our study. We found that CGA application reduced the frequency of rubbing and sneezing in AR mice in a concentration-dependent manner that CGA concentration was negatively related to the times of rubbing and sneezing. However, 2.5 mg/kg Dex application had a slightly stronger inhibitory effect on the rubbing and sneezing of AR mice, in comparison with 200 mg/kg CGA. Still, our results verified that CGA had a similar effect as Dex on AR-related symptom.

AR referred to as type 1 or immediate hypersensitivity reaction is implicated with IgE production in early- and late-stage response, and recruitment of eosinophils and basophils and release of Th2 cytokines mucosal surfaces as late-stage response [35–37]. Histopathological examination in our study showed enhanced eosinophils infiltration in nasal mucosa of AR mice, while CGA application attenuated eosinophils infiltration. Wang et al.’s study demonstrated that IL-17–producing Th2 cells induced a massive production of mucin and profound goblet hyperplasia [38]. Fang et al.’s study exhibited an evident mucus production and increased number of goblet cells in OVA-induced nasal mucosa and Gallic acid treatment alleviated these manifestations [39]. In consistent with their findings, we also observed a multiplied goblet cells and overproduction of mucus at nasal mucosa of AR mice and CGA application significantly reduced the number of goblet cells and mucus secretion.

The regulation of the differentiation and migration of Th17 is critical to the process of inflammation, infection, and autoimmunity [40]. Th2 over-differentiation in AR is associated with Th17/Treg cell imbalance [41]. ROR-γt can serve as a transcription factor that is vital to the effector function and differentiation of Th17 cells [42]. ROR-γt overexpression leads to IL-17 production [43]. Meanwhile, increased IgE level was discovered to indicate a high severity of AR [44,45]. Xu et al.’s study showed that generation of OVA-induced IgE was repressed, while FOXP3/Treg cell differentiation was promoted by tangeretin administration [28]. In accordance with these findings, our study demonstrated conspicuously up-regulated levels of ROR-γt, IL-17 as well as IgE in serum of AR mice, whereas CGA application repressed the up-regulated levels of these indices in AR mice. Our finding indicated that the anti-AR effect of CGA may be closely related to the inhibition of TH17 differentiation. IFN-γ, a Th1 cell cytokine can suppress Th2 cell proliferation to affect Th1/Th2 balance and induce macrophage production [46,47]. Increased level of IFN-γ contributes to shifting from Th2 to Th1 [48]. Fang et al.’s study demonstrated an increased level of IFN-γ in OVA-induced mice after treatment for AR [39]. Similarly, our study found an elevated serum level of IFN-γ in the presence of CGA, affirming the anti-AR effect of CGA.

In order to ascertain our surmise that CGA affected Th17 differentiation to alleviate AR-related symptom, the generation of CD4^+^IL-17^+^Th17 cells were assessed during AR. CD4^+^IL-17^+^Th17 cells were once discovered to induce autoimmune disorders [49]. Xiang et al.’s study showed that sema3A which can exert immunoregulatory effect pulled down the raised percentage of CD4^+^IL-17^+^Th17 cells and inhibited allergic inflammation in OVA-induced mice [50]. Likewise, we found a high ratio of CD4^+^IL-17^+^Th17 cells to CD4^+^T cells in RA mice, while CGA application lowered this ratio. Meanwhile, the facilitated mRNA and protein expression of ROR-γt and IL-17 were also suppressed by CGA application. Our finding verified that CGA inhibited the differentiation of CD4^+^T into Th17 cells to alleviate AR-related symptom.

Conclusively, gavage of CGA in AR mice alleviated AR-induced behaviors of nose rubbing and sneezing and histopathological abnormalities of eosinophils infiltration, mucus secretion, and goblet cell hyperplasia by suppressing Th17 differentiation. The discovery of our research indicated that CGA could be used as a potential candidate for the treatment of AR.

## Abbreviations

AR: allergic rhinitis
CGA: chlorogenic acid
*C*_t_: threshold cycle
Dex: dexamethasone
FOXP3: Forkhead Box P3
GAPDH: glyceraldehyde-3-phosphate dehydrogenase
HRP: Horseradish Peroxidase
IFN: interferon
Ig: immunoglobulin
IL: interleukin
OVA: ovalbumin
PAS: Periodic acid–Schiff
PBS: phosphate buffer saline
pTreg: Peripheral Regulatory cells
qRT-PCR: quantitative reverse-transcription polymerase chain reaction
ROR: retinoic acid-associated nuclear orphan receptor
TBST: Tris Buffered Saline and 1% Tween 20
Th: T helper cell
